# Use of Information Entropy and MACBETH Methods in the Replacement Rates of Multicriteria Methods: A Systematic Review of the Literature

**DOI:** 10.3390/e28080892

**Published:** 2026-08-08

**Authors:** Francine da Silva Borges, André Andrade Longaray

**Affiliations:** 1 Postgraduate Program in Computational Modeling, Federal University of Rio Grande, Rio Grande 96203-000, Brazil; francinesborges@gmail.com; 2 Institute of Economic, Administrative and Accounting Sciences, Federal University of Rio Grande, Rio Grande 96203-000, Brazil

**Keywords:** MACBETH, information entropy, substitution rates, multicriteria methods, MCDM/MCDA

## Abstract

Advances in the decision-making field and the growing adoption of hybrid mathematical methods, combined with practitioners’ and managers’ interest in more efficient systems, have intensified the search for improved combinations of mathematical modeling capable of supporting robust, effective decision processes. In this context, it becomes possible to incorporate different methods for assigning substitution rates in multicriteria methods, thereby reducing uncertainty in the application of such models. The purpose of this study was to examine research that applies the MACBETH method (measuring attractiveness by a categorical-based evaluation technique) in combination with other weighting techniques, particularly the information entropy method. The literature mapping evaluated the evolution of studies on this topic, the temporal progression of research, the most productive authors, and the journals most aligned with the theme while also identifying the techniques and sectors in which they are applied. The review further highlighted benefits, limitations, and future challenges and developments in the field. The research was conducted through a systematic literature review, with analysis conducted using a bibliometric approach complemented by a meta-synthesis. The findings reveal a growing number of publications in recent years, along with the main techniques combined for assigning substitution rates. The results also highlight the sectors of application, leading journals, and emerging trends in the domain of hybrid multicriteria methods. Given the limited research on this topic, this is considered a valuable contribution.

## 1. Introduction

In decision-making processes, decision-makers must evaluate different criteria and scenarios to identify the optimal solution that aligns with their needs and preferences. From the perspective of multicriteria decision-making, applications are diverse, with documented use in manufacturing, logistics distribution, healthcare, sales, enterprise resource planning, and business intelligence [1]. To evaluate such situations, multicriteria methods are employed to represent decision-maker preferences through an aggregation function.

Accordingly, different problem types may be addressed, such as selecting the best alternative (Pα), classifying alternatives as good or bad (Pβ), ranking alternatives (Pγ), and describing alternatives (Pδ), with the possibility of combining these within a single decision process [2,3]. Methods such as AHP, TOPSIS, and MACBETH are typically associated with Pα and Pγ problems, while the ELECTRE and PROMETHEE families are suited to Pα and Pβ problems [4].

In the decision-making process, both intra- and inter-criterion evaluation may occur. Intra-criterion evaluation concerns the assessment of each alternative relative to each criterion, enabling the construction of value functions and the development of an impact matrix or evaluation model [5]. Inter-criterion evaluation, by contrast, involves comparing alternatives through an overall value or score, with or without explicit pairwise comparisons [5].

Compensation in multicriteria aggregation procedures is commonly understood as allowing gains in one criterion to offset losses in another [6]. In this sense, weighting methods serve as reliable mechanisms for ensuring robust, accurate decisions, particularly in multicriteria contexts [7], where they contribute to fairer value attribution. The replacement rate, or weighting as it is also called, reflects the decision-maker’s judgment in numerical terms.

Among the various weighting methods described in the literature are the rank-order centroid method [8], simple mean [9], entropy [10], fuzzy entropy [11], and swing weighting [12]. Different formulations of entropy include thermodynamic entropy [13], information entropy [14], Tsallis entropy [15], nonadditive entropy [16], and Boltzmann–Gibbs entropy [17]. When combined, hybrid weighting proposals can improve the stability, sensitivity, and adaptability of decision models [7].

Several studies demonstrate these applications. Dehdasht, Ferwati, Zin and Abidin [18] employed TOPSIS and information entropy approaches to assess Environmental Sustainability Governance (ESG) factors in the Malaysian construction sector, analyzing key factors associated with the successful, sustainable implementation of lean construction. Roszkowska, Filipowicz-Chomko, Łyczkowska-Hańćkowiak and Majewska [19] integrated Mahalanobis distance with information entropy to develop the extended Hellwig method (H_EM), which aims to determine the importance of evaluated criteria and the relationships among them. The method was validated by examining educational progress in the EU in 2021 in relation to Sustainable Development Goal No. 4 of the 2030 Agenda. Ozdilek [20] combined information entropy with price, cost, and revenue estimates in real estate valuation, significantly improving the accuracy of value measurement and reducing errors in economic decision-making.

Given the existence of different combinations of multi-criteria methods to improve decision-making, making it more robust and realistic, this RSL seeks to understand which possible methods are already used in assigning replacement rates and how progress can be made in this area.

Considering these aspects, the present investigation is justified in terms of both importance and relevance [21]. It is necessary to analyze methods capable of improving the performance of multicriteria methods, particularly those that contribute to greater methodological rigor and reduced uncertainty. This study also meaningfully contributes to research by conducting a systematic literature review (SLR) that integrates the MACBETH method (measuring attractiveness by a categorical-based evaluation technique) with the information entropy technique, aiming to map existing studies and identify potential research gaps. This effort aligns with Mozumdar and Cinelli [22], who emphasized the limited use of weighting methods combined with MCDM/MCDA in nature-based solutions supported by computational applications. Furthermore, subjective methods in the weighting stage are difficult to express the effectiveness in evaluating the criteria, although they can handle ambiguous and imprecise information. Objective methods, on the other hand, perform this stage using real data, greatly favoring this combination, that is, extracting the best of each method in a hybrid model.

In light of these observations and the diverse contexts in which multicriteria methods are applied, SLR serves as a tool to address the following research questions:

RQ1: Which additional mathematical weighting techniques have been combined with MACBETH?
RQ2: How do these techniques intervene in the MACBETH weighting process?
RQ3: What are the benefits and limitations of the combinations identified in RQ1?
RQ4: In which sectors have the methods described in RQ1 been applied?

Accordingly, the objectives of this research are to

- Identify the weighting techniques used alongside MACBETH to determine substitution rates, thereby clarifying which tools in the literature have been combined with this subjective multicriteria method (MACBETH);
- Determine the type of intervention proposed by each study in the assignment of weights and the manner in which this intervention is carried out;
- Evaluate the functionality of these combinations by examining their positive and negative aspects;
- Propose future directions for advancing methodological combinations in the assignment of weights and determination of substitution rates under the MACBETH framework.

The next section presents the research methods, detailing the procedures for data collection and analysis, including the criteria for study inclusion, exclusion, selection, and evaluation. Section 3 presents the results, including the bibliometric analysis and the proposed meta-synthesis. Section 4 discusses the main results and outlines future research directions. Section 5 concludes the article with final considerations based on the evidence presented.

## 2. Materials and Methods

This exploratory investigation analyzed the application of multicriteria methods combined with information entropy. To map the study context, an SLR was conducted, supported by bibliometric analysis and meta-synthesis. The review followed the 27 steps of the PRISMA protocol (Preferred Reporting Items for Systematic Reviews and Meta-Analyses). The bibliometric analysis was guided by Bradford [23], Zipf [24], and Lotka [25].

Considering initial searches for terms such as information entropy, MACBETH, and other multi-criteria methods, the scarcity of combining these techniques was observed. Therefore, it was decided to proceed with a more comprehensive search for terms, investigating the use of entropy and other weighting techniques, along with MACBETH. To this end, three thematic areas were structured.

The search strategy was structured around three axes. Axis 1 focused on the theme of multicriteria decision-making and its synonyms. Axis 2 addressed information entropy and related terms. Axis 3 incorporated the theme of MACBETH and similar expressions, with an emphasis on substitution rates and weighting procedures.

The search was conducted on 15 September 2025, using databases indexed in the Capes journal portal. The consulted databases included ASP-EBSCO, IEEE, ScienceDirect, Scopus, and Web of Science, and the search was limited to publications in English. Because the purpose of the review was to not only map the context but also identify research gaps, no restrictions were placed on publication type.

The search string was composed exactly in this way, considering (“Axis 1”) AND (“Axis 2”) AND (“Axis 3”). From these combinations, it was possible to obtain a total of 71 publications, according to the databases identified in Table 1.

**Figure 1:**
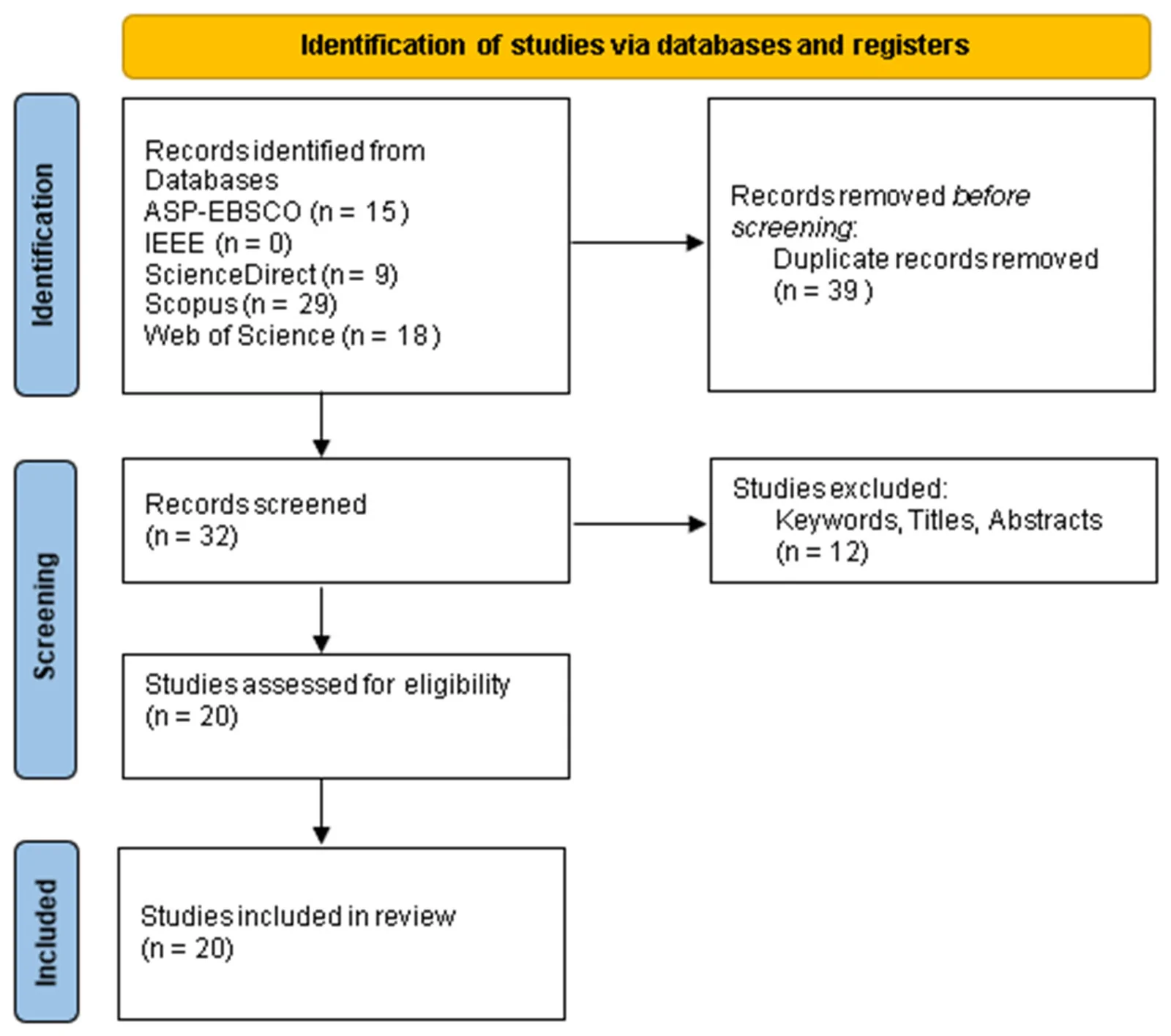
Study selection flowchart. Source: Adapted from Page et al. [27].

After data collection, the publications were organized using Zotero [26]. A total of 39 duplicates were removed, resulting in 32 unique articles for evaluation. This initial portfolio underwent a filtering process based on reading keywords, titles, and abstracts to assess alignment with the study’s theme. After this step, 11 articles were excluded, leaving 20 publications for the final analysis portfolio. At this stage, the necessary information was extracted to address the research objectives. Figure 1 illustrates these steps.

Figure 1 illustrates the process of conducting the SLR according to the PRISMA protocol. It presents the stages of identifying records in the databases, removing duplicates, and filtering publications based on the congruence of keywords, titles, and abstracts with the research theme. The figure also demonstrates the inclusion of articles selected for refined analysis, forming the final portfolio used for data extraction and interpretation.

The illustrations were produced using raw data from the portfolio and with the aid of code implemented in Python version 3.12.13, operationalized in Google Colab. Libraries such as pandas, matplotlib, networkx, wordcloud, textblob, seaborn, and plotly, among other language functions, were used to construct the graphs and images included in the analysis results. These libraries are capable of verifying the frequency of distributions, as well as determining, through graphs, the connections between elements.

The articles selected in the filtering process underwent full reading, and it was verified whether there was a weighting technique different from that used in MACBETH. For this purpose, the analysis portfolio, composed of the six remaining works, was selected.

## 3. Results

Following the identification, screening, and inclusion stages, it became possible to analyze both the raw portfolio and the refined analysis portfolio. The sections that follow describe the bibliometric analyses of both portfolios, as well as the meta-synthesis conducted on the final set of studies.

### 3.1. Bibliometric Analysis of the Raw Portfolio

**Figure 2:**
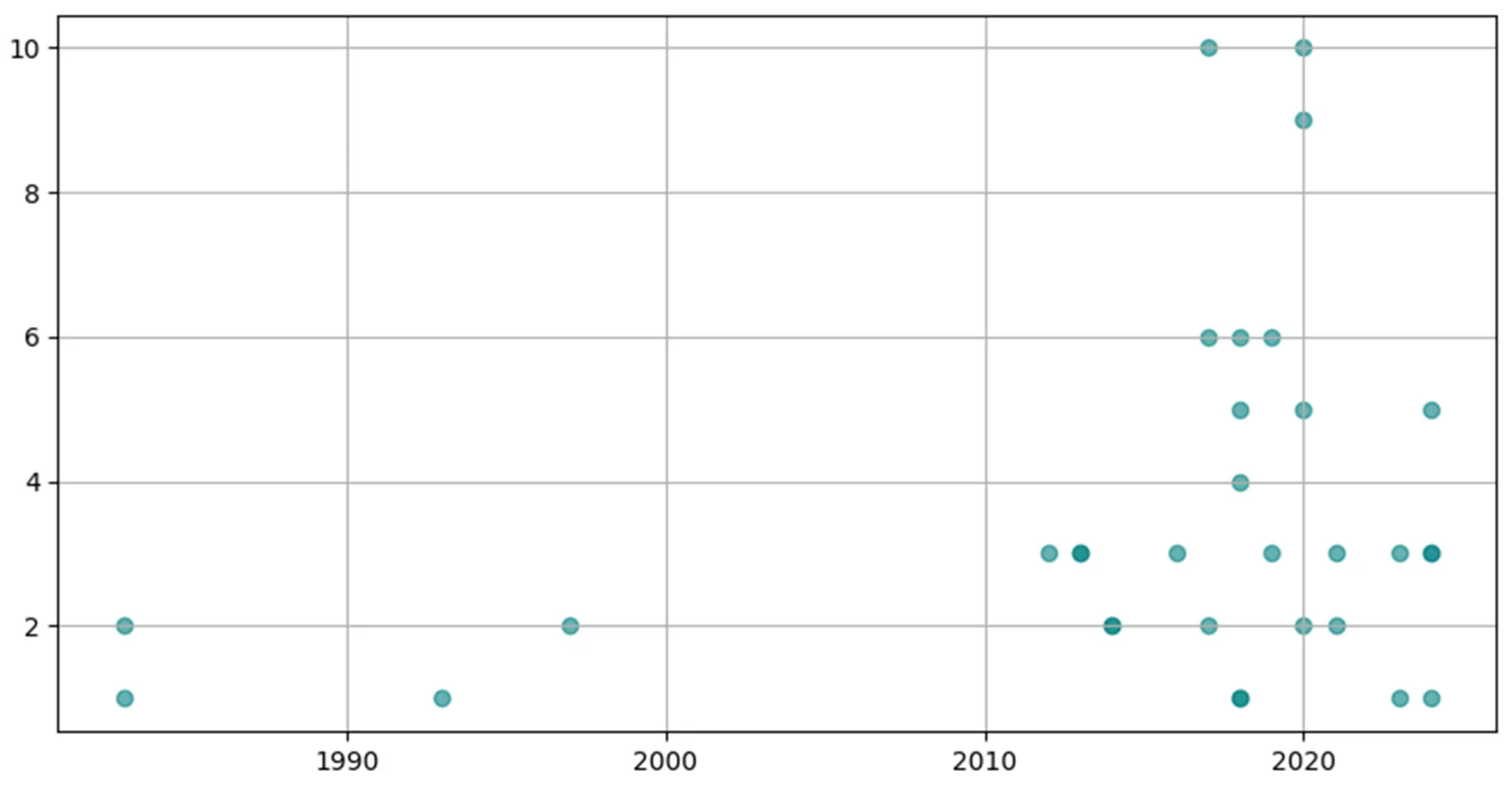
Concentration of publications by year. Source: Authors (2026).

The temporal evolution of the collected data began in the 1990s, showing a stable trend between 2000 and 2010, followed by a period of increasing fluctuation approaching the 2020s. The number of publications ranged from a minimum of one to a maximum of five per year, as shown in Figure 2. This pattern aligns with the concentration of author publications over time. Figure 2 presents the grouping of authors and the distribution of their publications, revealing a notable concentration around the year 2020. This outcome is consistent with the temporal evolution, indicating that most publications combining information entropy with the MACBETH method emerged during this period. We can also infer that this concentration spans a window of approximately five years before and after 2020.

**Figure 3:**
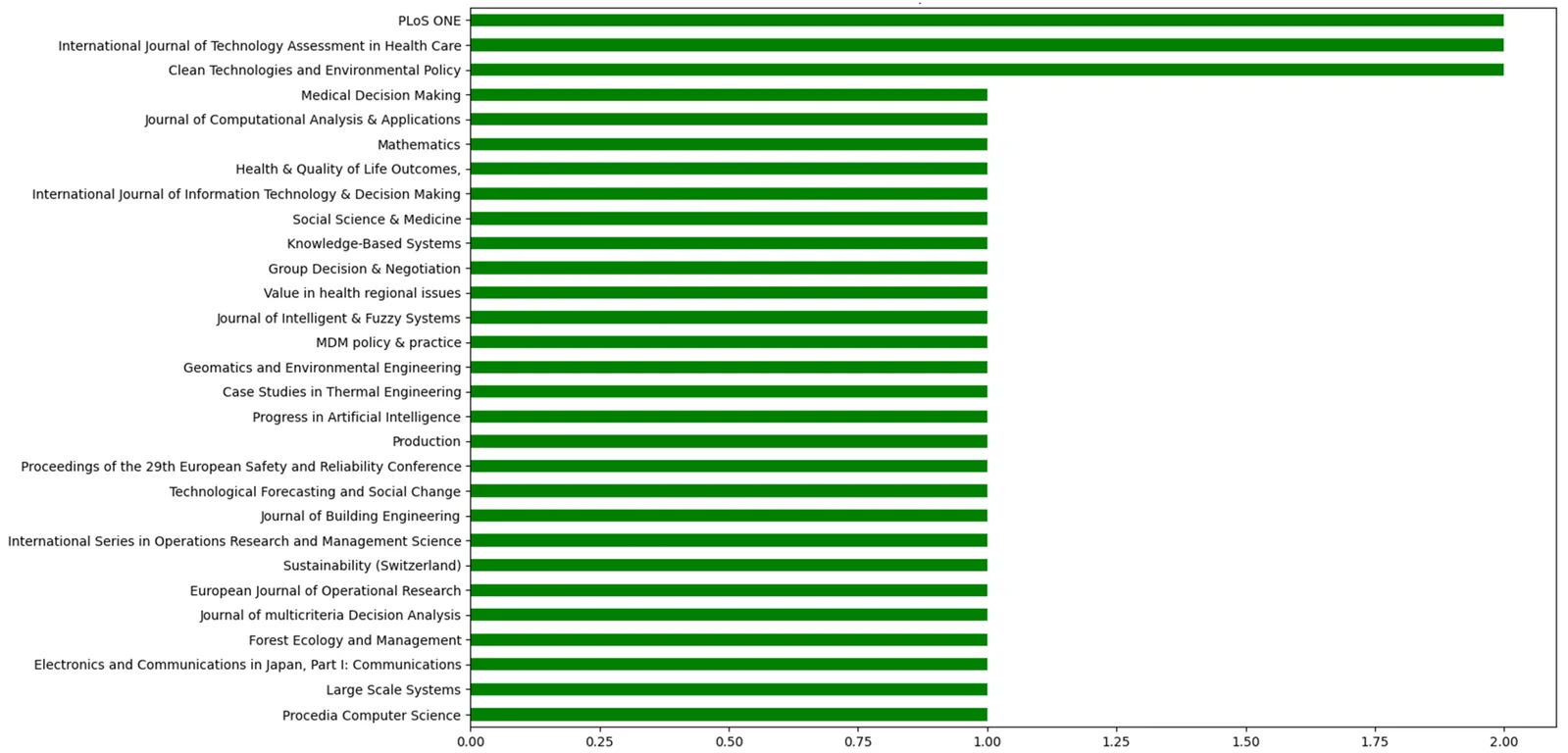
Number of publications per journal. Source: Authors (2026).

Regarding journal productivity, three journals—PLoS One, the International Journal of Technology Assessment in Health Care, and Clean Technologies and Environmental Policy—each published two articles within the portfolio. All other journals contributed one publication each, as shown in Figure 3. This distribution corresponds to an average of 1.10 articles per journal. The journals represent fields such as technology, mathematics, and health, underscoring the diverse applications of operational research and multicriteria decision-making.

**Figure 4:**
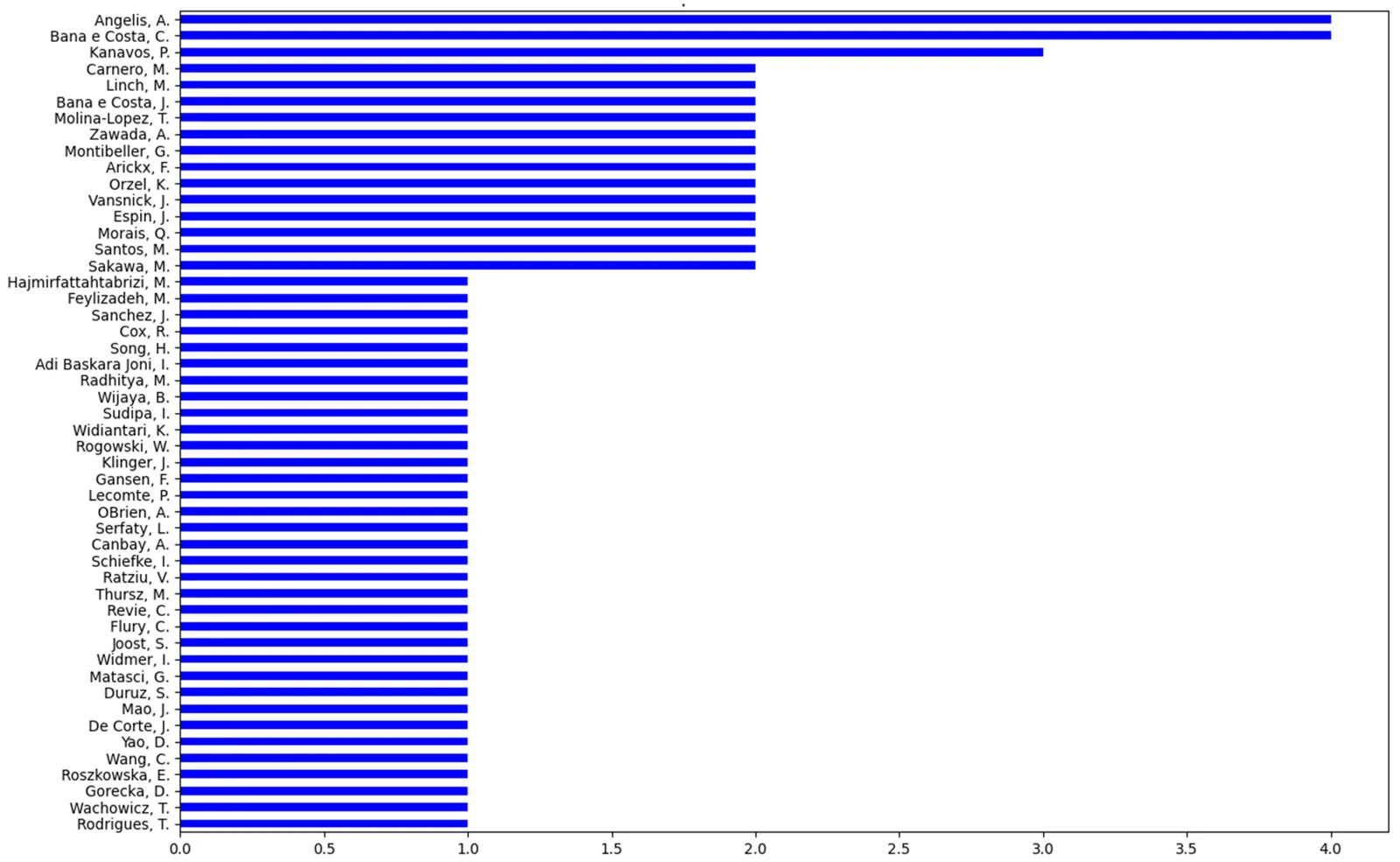
Authors and their respective publication counts. Source: Authors (2026).

Author productivity was also assessed based on the number of publications, as shown in Figure 4. The most prominent authors were Angelis, A., and Bana e Costa, C., with four publications, and Kanavos, P., with three publications, followed by Carnero, M., Linch, M., Bana e Costa, J., Molina-Lopez, T., Zawada, A., Montibeller, G., Arickx, F., Orzel, K., Vansnick, J., Espin, J., Morais, Q., Santos, M., and Sakawa, M., with two publications. The remaining 34 authors contributed one publication each. The portfolio comprises 32 publications and a total of 50 individuals, resulting in an average of 3.53 authors per article.

**Figure 5:**
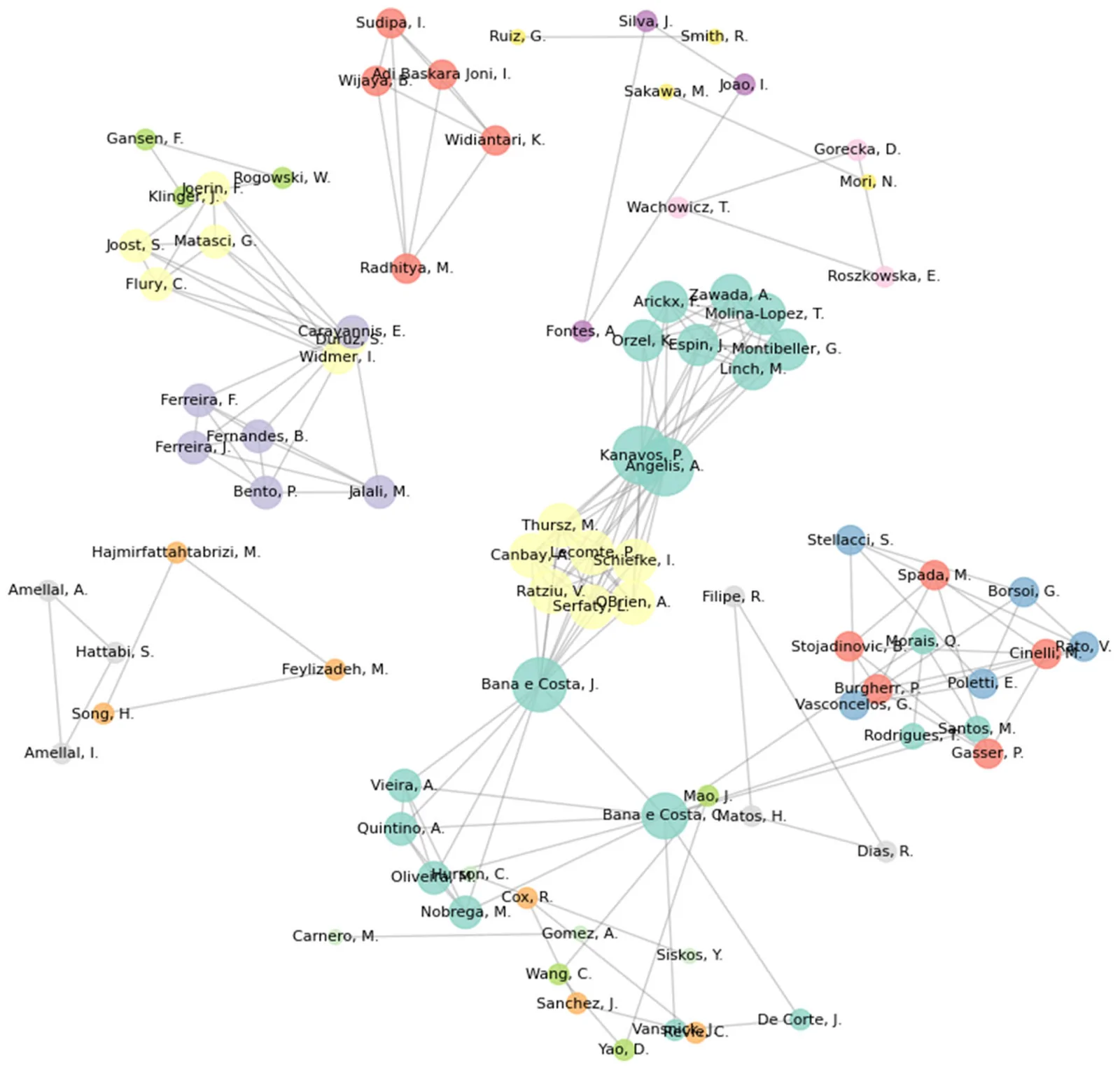
Coauthor network in the raw portfolio. Source: Authors (2026).

In addition to these analyses, the coauthorship network was examined through graphical analysis, as presented in Figure 5. The network is represented by nodes indicating author centrality and colors denoting communities connected through coauthorship links. Each author is connected to their coauthors, forming a network that reflects collaborative relationships. Larger nodes represent authors with a higher number of publications, while authors who published together are grouped in the same color, visually highlighting collaborative clusters.

These authors form a large cluster, followed by five smaller clusters that show no interconnections. The larger cluster represents multiple communities interacting across different publications. It is notable that the seminal author of the MACBETH method, Bana e Costa, C., appears at its center. Bana e Costa, J., functions as a bridge between the community linked to Bana e Costa, C., and the community formed by Angelis, A., and Kanavos, P. Two additional clusters exhibit interconnections among their authors, indicating an active exchange of experience and collaboration. Three other clusters appear isolated, representing the absence of collaborative links with the remaining groups. The coauthorship network can also be interpreted through author centrality. The centrality indices assigned to the main authors were as follows: Angelis, A.—0.188; Kanavos, P.—0.188; Bana e Costa, J.—0.165; and Bana e Costa, C.—0.118.

**Figure 6:**
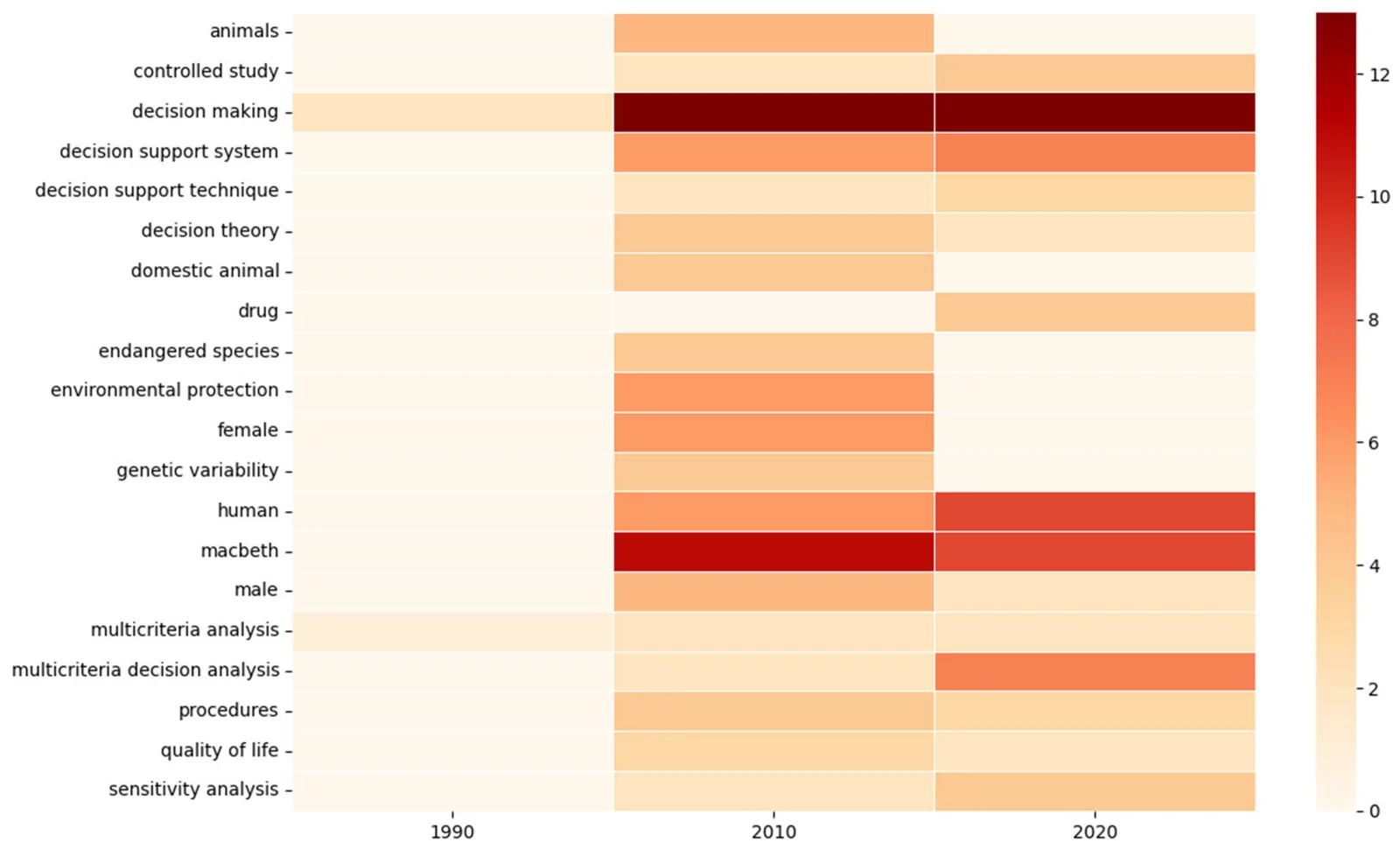
Heatmap of keyword frequency by decade. Source: Authors (2026).

The analysis also examined the keywords listed by authors in the selected articles. Two heatmap graphs were generated to evaluate keyword frequency over time, focusing on the 1990s, 2010s, and 2020s. Figure 6 shows that the keyword “decision making” has the highest frequency across all decades, first emerging in the 1990s, increasing in the 2010s, and reaching its peak in the 2020s. The keyword “macbeth” is the next most prominent, with its greatest visibility in the 2010s and a slight decline in the 2020s. “Decision support system” begins to stand out in the 2010s and grows further in the 2020s, as does “multicriteria decision analysis.” The remaining terms show more uniform frequency and are not notably prominent.

**Figure 7:**
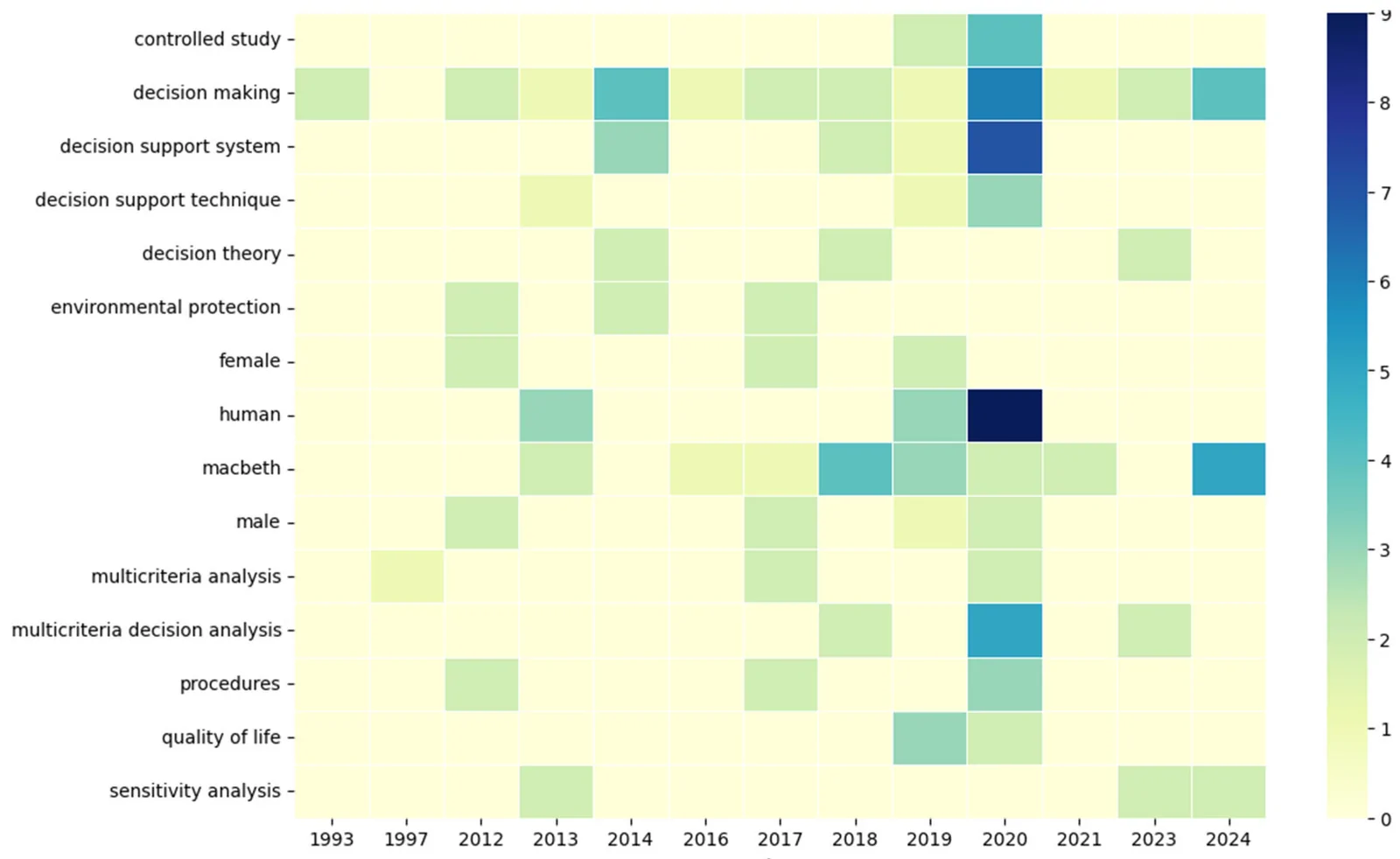
Heatmap of keyword frequency by year of occurrence. Source: Authors (2026).

Figure 7 presents the results by year of occurrence. The keyword “decision making” is consistently highlighted from 1993 to 2024, maintaining clear dominance over the other terms. As we can see in the graph, the highlight in relation to the other terms is noticeable. This is followed by “decision support system,” “multicriteria decision analysis,” and “human” in 2020; “macbeth” in 2018, 2019, and 2024; “quality of life” in 2019; and “controlled study,” “decision support techniques,” and “procedures” in 2020. The remaining terms appear at least once during the evaluated period.

Considering the terms extracted from the keywords, titles, and abstracts, diagrams were constructed to evaluate the frequency of these occurrences and assess the alignment of the selected articles with the search strategy used in the SLR. In these diagrams, words with higher frequency are larger.

**Figure 8:**
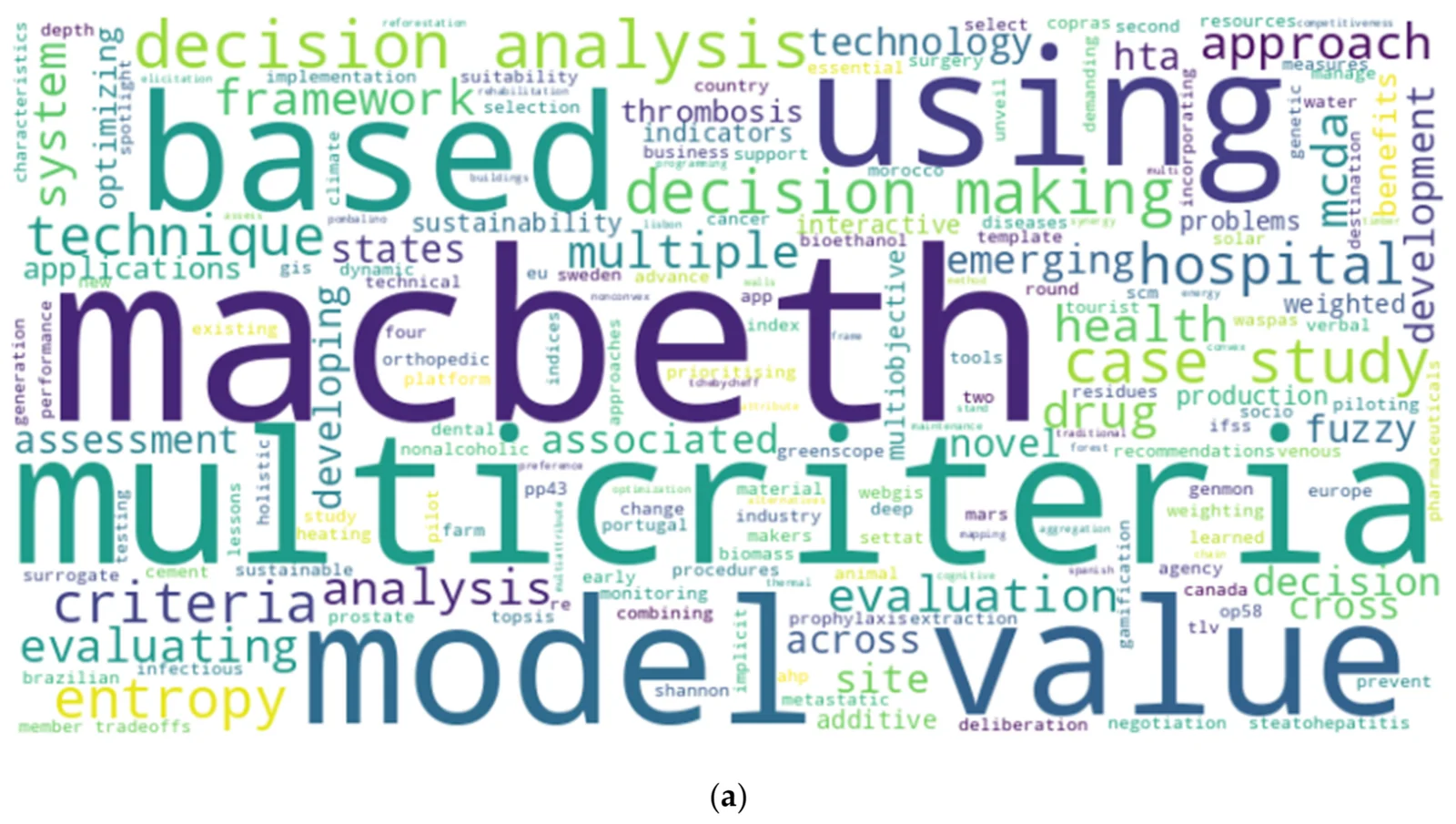

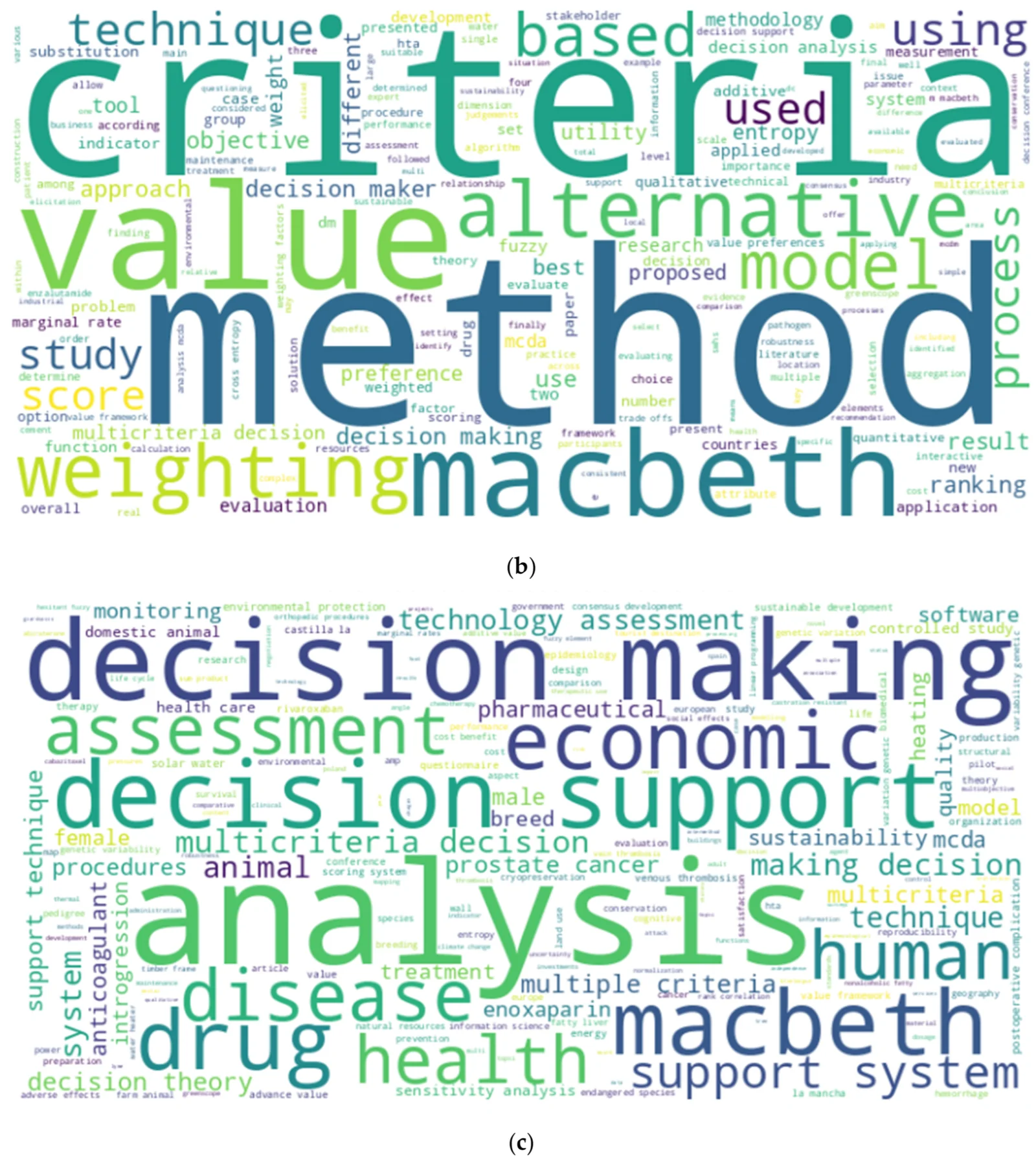
Word-cloud frequency diagram grouped by (**a**) titles, (**b**) abstracts, and (**c**) keywords. Source: Authors (2026).

Figure 8a presents the diagram produced from the recurring words in the titles of the articles in the raw portfolio. Terms such as “macbeth,” “multicriteria,” “model,” “using,” and “based” are clearly identifiable, followed by “decision analysis” and “decision making.” These terms closely reflect those used in the search string.

Similarly, the diagram for the abstracts, shown in Figure 8b, highlights the terms “method” and “value,” followed by “macbeth.” These three terms appear with more prominence than the others and remain consistent with the search string.

The diagram constructed from the keywords, presented in Figure 8c, highlights “analysis,” “decision making,” and “decision support,” followed by “economic,” “system,” “assessment,” and “macbeth.” The recurrence of these terms across the diagrams reinforces their alignment with the search strategy used in the databases. This consistency observed in the results can be associated with the keywords chosen for the search strategies, which seek to represent the research topic in a broad and coherent way.

**Figure 9:**
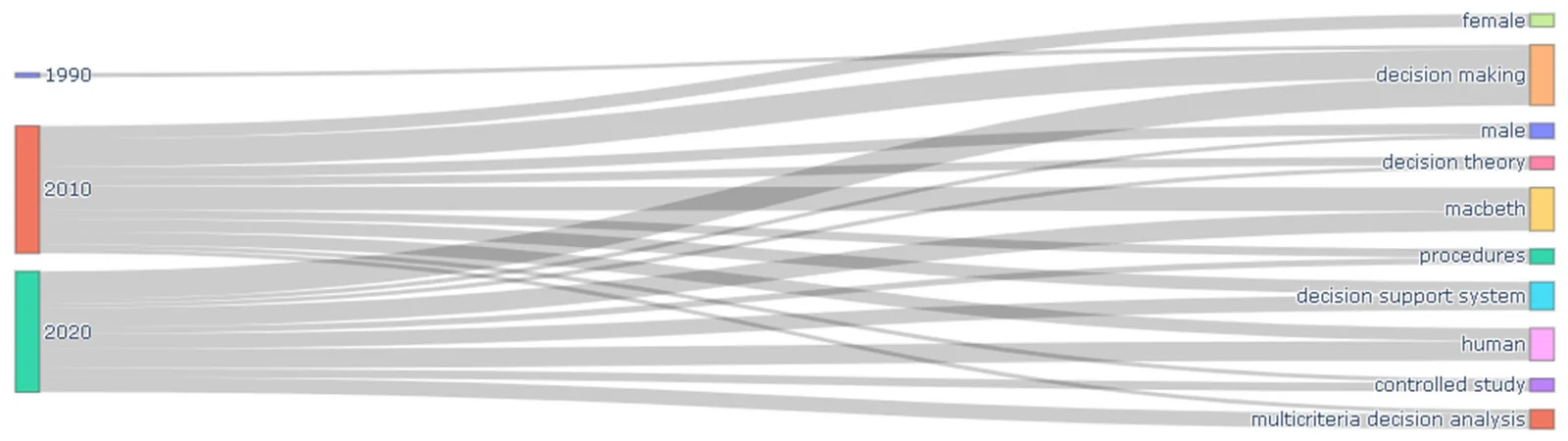
Keyword flow diagram by decade. Source: Authors (2026).

A Sankey diagram was also developed to illustrate the flow of keywords by decade. Figure 9 displays the 1990s, 2010s, and 2020s on the left, with the corresponding keywords on the right, connected by flow lines and represented by colored quadrilaterals. The diagram shows an increase in publications in the 2010s and 2020s compared with the 1990s, along with the evolution of keyword prominence across decades.

The quadrilateral size reflects the frequency of each term. “Decision making” emerges in the 1990s and gains increasing prominence in the 2010s and 2020s. The remaining highlighted terms appear predominantly in the 2010s and 2020s, with particular emphasis on “macbeth” and “human.”

### 3.2. Summary of Bibliometric Results

The raw portfolio made it possible to identify the main journals publishing in the field of operations research, particularly those presenting studies on multicriteria methods. The results also indicated that the keywords used in the search strategy were well aligned with the retrieved publications, revealing thematic coherence. Furthermore, the temporal evolution of the literature indicated a growing prominence of operations research in recent decades, especially in decision-making, reinforcing the expansion of this field over time.

The analysis portfolio consists of articles that apply the MACBETH multicriteria method in combination with other mathematical techniques for assigning substitution rates. Six studies were identified as capable of answering the research questions and revealing the methodological choices made by the authors. These studies permit an examination of the contexts in which the methods were applied, the techniques incorporated into the substitution rates, and the benefits and limitations associated with these methodological combinations.

### 3.3. Analysis Portfolio Assessment

Meta-synthesis was selected as the method for examining the analysis portfolio, enabling a more detailed and rigorous evaluation. After the filtering process, 20 articles remained aligned with the research theme and were selected for full reading. From these readings, the relevant information was extracted according to the research objectives, regardless of the specific focus of each study. Because the review also sought to identify potential research gaps, some articles required deeper examination to determine their relevance.

Table 2 presents the 20 articles selected for complete analysis. The first column lists the identification index, followed by the authors, article title, and journal of publication.

The readings provided preliminary insights into the sectors in which the studies were applied. These included business and management (ID_1, ID_8, ID_20), health (ID_2, ID_5, ID_6, ID_9, ID_10), pharmacy (ID_3, ID_11), education (ID_4), theoretical modeling (ID_7), civil engineering (ID_12, ID_16, ID_18, ID_19), alternative energy (ID_13, ID_14, ID_17), and tourism (ID_15). These findings illustrate the broad applicability of multicriteria methods—particularly MACBETH—across diverse domains.

However, it was observed that 14 of the 20 articles did not simultaneously address the research questions. These articles were therefore excluded from the final analysis, leaving six studies that fully met the guiding criteria.

### 3.4. Addressing the Research Questions

From the six selected articles, an in-depth analysis was carried out to extract the information necessary to answer the research questions, as described in the introduction of this article.

To answer RQ1, the studies were examined to identify the techniques used alongside MACBETH. Articles ID_1, ID_2, ID_4, ID_9, ID_13, and ID_18 were found to employ mathematical techniques different from the original MACBETH weighting procedure.

In response to RQ2, the techniques used for assigning weights are summarized in Table 3. The authors mention several possible methods, including the centroid method, simple mean, simulated probabilistic distribution, information entropy, information entropy combined with fuzzy numbers, MAUT, and UTA.

All of these techniques intervene in the weighting of criteria. Some studies, such as Carnero et al. [11] and Singh [10], include additional algorithms to improve robustness, including TOPSIS and WASPAS.

Regarding the benefits and limitations addressed in RQ3, Table 4 summarizes the findings for studies that apply alternative weighting techniques in combination with MACBETH.

ID_9 is a conference abstract. As such, the available information is succinct and lacks the level of detail observed in the other studies, providing only essential descriptive elements.

To answer RQ4, the sectors in which the articles intervening in the MACBETH method were applied were identified. These applications illustrate the breadth and versatility of multicriteria methods across domains. The five studies span diverse areas such as business and management, health, education, alternative energy, and civil engineering, as summarized in Table 5.

## 4. Discussion, Overview, Challenges, and Future Trends

The studies reveal that the field continues to evolve. This evolution is accompanied by the increasing use of computational techniques, enabling the operationalization of more robust and diverse approaches for assigning substitution rates. It is also evident that these techniques are expanding across sectors and research contexts, reinforcing the versatility of multicriteria methods.

### 4.1. Summary of Findings

Sudipa et al. [8] examined a problem in the area of business and management involving 150 entrepreneurs. The authors applied MACBETH combined with the centroid method to assign criterion weights. Five criteria—profit, capital, promotion, competitors, and business prospects—were assessed across five commercial alternatives: cake shop, flower shop, cake-utensil shop, electronics store, and stationery store.

Cox et al. [9] assessed the emergence of diseases under climate-change scenarios. The study used MACBETH, with weights assigned through the simple average of values derived from probability or importance classifications. A second weighting approach used probabilistic distributions generated through simulation. The simple-average method produced a more direct ranking, whereas probabilistic distribution captured uncertainty in expert judgment and revealed variation in intermediate rankings. Both approaches yielded similar overall results, with differences concentrated in mid-range alternatives.

Carnero [11] selected a gamification tool for academic use through multicriteria analysis. The study combined MACBETH with fuzzy information entropy and fuzzy TOPSIS and validated the results through sensitivity analysis and comparison with rankings generated by VIKOR, PROMETHEE II, and ELECTRE III.

Angelis et al. [12] conducted a multicriteria evaluation of therapeutic alternatives for prostate cancer using MACBETH with swing weighting. The criteria included therapeutic impact, safety profile, level of innovation, and economic impact. Therapeutic impact was the most important criterion, and sensitivity analysis was implemented to assess model robustness.

Singh [10] optimized solar water-heating systems by integrating MACBETH, information entropy, and WASPAS. Information entropy was used to derive objective weights from the dispersion of values in the decision matrix. Hurson and Siskos [40] combined MAUT, MACBETH and UTA to evaluate metro-line planning in Athens. MAUT was used to elicit explicit trade-offs, MACBETH supported qualitative comparisons among criteria and alternatives, and UTA was applied to infer value functions and weights from global rankings.

#### Weighting Methods

Among the various weighting methods identified in the analysis portfolio resulting from the SLR, the following were applied in the reviewed studies: rank order centroid (ROC), MAUT and UTA, simple mean, simulated probabilistic distribution, information entropy, and fuzzy information entropy. Their applications are described below.

ROC determines the priority of each criterion by assigning decreasing weights from the first to the nth criterion [8]. The method assumes that true weights are equally likely to lie anywhere within the feasible polytope, making centroid coordinates the best available approximations.

The simple mean approach calculates criterion weights as the average of expert assessments [9]. In the referenced study, experts provided qualitative likelihood ratings (do not know = 0, not likely = 0.1, very likely = 0.3, probable = 0.5, very likely = 0.7, extremely likely = 0.9). Importance ratings followed a similar qualitative-to-quantitative mapping. These fixed numerical scores were then used to compute the criterion weights within the model.

Simulated probabilistic distribution was used based on expert opinion [9]. The authors used the ModelRisk Excel add-on to generate a random number from the discrete likelihood distribution. The distribution used the interval from 0.1 to 1. For the weight of “unlikely,” in the interval of 0.01 and 0.19, a random number was defined in this interval. In all, 10,000 simulations were carried out to determine the weights of the criteria.

Singh [10] applied information entropy to determine the weights for selecting solar-collector alternatives. The method was integrated with WASPAS and MACBETH to identify optimal combinations of operational and design parameters that maximize performance across all criteria.

Fuzzy information entropy was used alongside MACBETH to determine subjective and objective weights simultaneously [11]. The author highlighted the methodological innovation of this combination. The study compared information entropy and exponential information entropy to validate the proposal. The method was integrated with TOPSIS and benchmarked against other approaches. Fuzzy information entropy incorporates a discrete random variable defined over a finite set, following the same steps as classical entropy. In that study, weight aggregation was performed using α = 0.5.

The final proposal incorporated MAUT and UTA with a revised MACBETH procedure. The method recommends evaluating marginal value functions and weights using MACBETH. If this is not feasible, MAUT is applied, and if challenges persist, the UTA disaggregation method is used [40].

Based on the identified studies, it can be stated that the main advantages of applying the MACBETH method lie in the systematic way it structures judgments. Furthermore, it allows for the transformation of qualitative assessments into consistent scales. However, it still heavily depends on the participation of experts and the consistency of judgments, which allows for subjectivity in weight assignment. However, this dependence can be minimized through a hybrid combination of methods, particularly more objective ones that use real data as inputs, such as information entropy, bringing greater objectivity and robustness to decision-making. It should be noted that the hybrid combination of methods allows for the exploitation of the best characteristics of each. In addition, it should be observed that the method is little explored in the literature, especially regarding improvements in the assignment of replacement rates and hybrid combinations with other methods, which could bring it closer to implementation in real-world scenarios.

### 4.2. Research Gaps

Based on the studies analyzed in the portfolio, several research gaps are pointed out and identified by the portfolio’s own authors. Therefore, it can be seen that these limitations can provide valuable directions for future research. Table 6 summarizes these gaps, organized according to each study and the corresponding gaps highlighted by the authors.

### 4.3. Challenges

Several challenges emerge from the analysis. One concerns the integration of multicriteria methods with more robust weighting techniques, particularly in dynamic or complex contexts. Another challenge relates to the generalizability of computational implementations, which may be restricted by the difficulty of replicating models or by geographic constraints that are often not incorporated into the modeling process.

The availability of reliable and accurate data, expert judgment, and questionnaire-based inputs is a critical foundation for multicriteria analyses. These elements are essential for the successful construction and development of the model; however, they can also become significant bottlenecks in the modeling process. When any of these components are weak, inconsistent, or incomplete, the quality of the results may be compromised.

Another challenge includes the implementation of models in free or low-cost software environments with accessible learning curves. This is compounded by the need for prior methodological knowledge and the high computational complexity of some models, which can increase the effort required for development and application.

Bias introduced by decision-makers during the evaluation of criteria or alternatives also poses a challenge. Weighting methods can significantly influence final results, and inadequate validation or lack of consistency checks may further compromise the reliability of multicriteria models. However, the evaluated scenario and the applied techniques must be considered, since the use of hybrid methods can make the problem more complex to solve and increase the computational cost involved.

### 4.4. Future Trends

Future research is likely to explore the inclusion of different weighting techniques to enhance robustness and result quality, particularly within the MACBETH framework and other multicriteria methods. Emphasis may be placed on decision-making supported by objective data, combined with validation procedures and consistency verification to assess model efficiency and effectiveness.

New techniques for assigning substitution rates may also be developed, particularly those integrating multiple multicriteria methods. The implementation of these methods in free and accessible computational environments may support the development of more complex models.

The use of more reliable data is expected to become increasingly relevant. Researchers may also focus on including more sensitive risk-related variables and considering indirect effects in their analyses.

## 5. Final Thoughts

This study explored research applying the MACBETH method together with information entropy for weighting substitution rates. SLR was conducted, supported by bibliometric analysis and meta-synthesis, allowing a comprehensive understanding of the research landscape and potential avenues for advancement.

The raw portfolio enabled the identification of the leading journals publishing work in operations research, particularly studies focused on multicriteria methods. The strong alignment between the search keywords and the retrieved publications confirmed the consistency and effectiveness of the search strategy. Furthermore, the temporal evolution of the literature indicated a marked increase in scholarly output in operations research in recent decades, particularly in the field of decision making.

Six studies were identified as capable of answering the research questions and clarifying the methodological choices made by the authors. These studies allow for an understanding of the application contexts, the techniques integrated into substitution rates, and the benefits and limitations associated with these combinations.

Based on the studies analyzed, it is noted that the use of hybrid approaches was employed to take advantage of the complementary characteristics of the methods. Bearing in mind that their choice depends on the characteristics of the problem and the issues involved, and understanding the diversity of applications, there is insufficient evidence to assert the superiority of one weighting method over another.

This study achieved its objective by identifying the alignment of search terms, the main contributing authors, the journals involved, and the temporal evolution of research. It also identified the different weighting techniques, various uses of information entropy, and diverse application scenarios of multicriteria methods.

Recognizing that search term alignment is critical for a well-conducted review, a potential drawback of this study is the possible omission of relevant terms not included in the search strategy. Coupled with the limited number of studies available for consultation. Future research may benefit from expanding the search vocabulary and integrating additional substitution rate techniques compatible with the MACBETH method.

Furthermore, it is highlighted that future studies may provide new interactions between multicriteria methods. Likewise, developing studies in the context of assigning replacement rates in the context of decision-making.

## Figures and Tables

**Table 1:** Search axes and data collection in the databases.

Axes	ASP—EBSCO	IEEE	Science Direct	Scopus	Web of Science
Axis 1—(“multicriteria” OR “multi-criteria” OR “mcdm” OR “decision making” OR “mcda” OR “decision analysis”)	15	0	9	29	18
Axis 2—(“entropy” OR “entropy weight method” OR “ewm” OR “weighting” OR “entropy weighting” OR “intracriteria rate” OR “Information Theory”)					
Axis 3—(“macbeth” OR “replacement rates” OR “rate of substitution” OR “substitution ratio”)					
Total	71				

Source: Authors (2026).

**Table 2:** Articles to refine from the analysis portfolio.

ID *	Authors	Title	Journal
ID_1	Sudipa et al. [8]	Dynamic Criteria Decision-Making Model for Business Development Recommendations Using Macbeth and Surrogate Weighting Procedures	*Journal of Computational Analysis & Applications*
ID_2	Cox et al. [9]	Multi-Criteria Decision Analysis Tools for Prioritising Emerging or Re-Emerging Infectious Diseases Associated with Climate Change in Canada	*PLoS ONE*
ID_3	Angelis, Thursz, Ratziu, O’Brien, Serfaty, Canbay, Schiefke, Costa, Lecomte and Kanavos [28]	Early Health Technology Assessment during Nonalcoholic Steatohepatitis Drug Development: A Two-Round, Cross-Country, Multicriteria Decision Analysis	*Medical Decision Making*
ID_4	Carnero [11]	Developing a Fuzzy TOPSIS Model Combining MACBETH and Fuzzy Shannon Entropy to Select a Gamification App	*Mathematics*
ID_5	Gansen, Klinger and Rogowski [29]	MCDA-Based Deliberation to Value Health States: Lessons Learned from a Pilot Study	*Health & Quality of Life Outcomes,*
ID_6	Angelis, Linch, Montibeller, Molina-Lopez, Zawada, Orzel, Arickx, Espin and Kanavos [30]	Multiple Criteria Decision Analysis for HTA across four EU Member States: Piloting the Advance Value Framework	*Social Science & Medicine*
ID_7	Bana e Costa et al. [31]	MACBETH	*International Journal of Information Technology & Decision Making*
ID_8	Górecka, Roszkowska and Wachowicz [32]	The MARS Approach in the Verbal and Holistic Evaluation of the Negotiation Template	*Group Decision & Negotiation*
ID_9	Angelis et al. [12]	OP58 Testing Of A Multiple Criteria Decision Analysis Value Framework With Decision Makers Across Europe	*International Journal of Technology Assessment in Health Care*
ID_10	Santos, Morais, Rodrigues and Bana e Costa [33]	PP43 MACBETH In Brazilian Hospital-Based HTA: Thrombosis Prophylaxis	*International Journal of Technology Assessment in Health Care*
ID_11	Angelis [34]	Evaluating the Benefits of New Drugs in Health Technology Assessment Using Multiple Criteria Decision Analysis: A Case Study on Metastatic Prostate Cancer With the Dental and Pharmaceuticals Benefits Agency (TLV) in Sweden	*MDM Policy & Practice*
ID_12	Amellal, Amellal and Hattabi [35]	Optimizing Selection of Sustainable Material-extraction Sites with GIS-based Decision-support Systems (AHP/MACBETH): In-depth Case Study from Settat, Morocco	*Geomatics and Environmental Engineering*
ID_13	Singh [10]	Entropy Weighted WASPAS and MACBETH Approaches for Optimizing the Performance of Solar Water Heating System	*Case Studies in Thermal Engineering*
ID_14	Fontes, João and Silva [36]	Multicriteria Evaluation of Biomass Residues in Portugal to Second Generation Bioethanol Production	*Production*
ID_15	Carayannis, Ferreira, Bento, Ferreira, Jalali and Fernandes [37]	Developing a Socio-Technical Evaluation Index for Tourist Destination Competitiveness Using Cognitive Mapping and MCDA	*Technological Forecasting and Social Change*
ID_16	Stellacci, Rato, Poletti, Vasconcelos and Borsoi [38]	Multi-Criteria Analysis of Rehabilitation Techniques for Traditional Timber Frame Walls in Pombalino Buildings (Lisbon)	*Journal of Building Engineering*
ID_17	Carnero and Gómez [39]	A Multicriteria Model for Optimization of Maintenance in Thermal Energy Production Systems in Hospitals: A Case Study in a Spanish Hospital	*Sustainability (Switzerland)*
ID_18	Hurson and Siskos [40]	A Synergy of Multicriteria Techniques to Assess Additive Value Models	*European Journal of Operational Research*
ID_19	Bana e Costa and Vansnick [41]	Applications of the MACBETH Approach in the Framework of an Additive Aggregation Model	*Journal of Multi-Criteria Decision Analysis*
ID_20	Bana e Costa et al. [42]	Collaborative Value Modelling in Corporate Contexts with MACBETH	*Procedia Computer Science*

Source: Authors (2026). * Identifying the items within the portfolio.

**Table 3:** Articles included in the final analysis portfolio.

ID *	Authors	Method	Mathematical Intervention
ID_1	Sudipa et al. [8]	MACBETH	ROC Method—Rank Order Centroid Method
ID_2	Cox et al. [9]	MACBETH	Simple Mean/Simulated Probabilistic Distribution
ID_4	Carnero [11]	MACBETH, Entropy, Fuzzy, and TOPSIS	Entropy Fuzzy
ID_9	Angelis et al. [12]	MACBETH	Swing Weight
ID_13	Singh [10]	MACBETH, Entropy, WASPAS	Entropy
ID_18	Hurson and Siskos [40]	MAUT, MACBETH, UTA	MAUT and UTA

Source: Authors (2026). * Identifying the items within the portfolio.

**Table 4:** Benefits and limitations identified in the reviewed studies.

ID *	Benefits	Limitations
ID_1	Integration of qualitative and quantitative criteria; reduced subjectivity in weight assignment; adaptability of the model to organizational contexts; strengthened support for decision-makers.	Need for sensitivity analysis; geographic constraints; dependence on questionnaire-based data.
ID_2	Systematic and transparent prioritization process; ability to incorporate heterogeneous information; adaptability for users with different expertise levels; ease of implementation in spreadsheets and M-MACBETH software (version 2.3.0. – 2010).	Dependence on data availability and expert judgment; high uncertainty in disease-related parameters; software cost and learning curve; inability to capture indirect climate-change effects; results sensitive to weighting structure; nonapplicable or unknown criteria may underestimate risks; absence of validation against a reference standard for disease emergence.
ID_4	Integration of MACBETH with fuzzy entropy methods reduces bias; MACBETH guarantees judgment consistency; fuzzy TOPSIS is intuitive, flexible, and effective under uncertainty; model stability confirmed through sensitivity analyses; comparative evaluation demonstrates reliability of rankings.	Potential evaluator bias; requirement for consensus across methods to finalize rankings; limitations of free software versions for implementation; risk of ranking reversal in TOPSIS when alternatives are added or removed; absence of judgment-consistency verification in some steps.
ID_9	Transparency, methodological systematization, and capacity to integrate diverse values and criteria.	Not reported.
ID_13	Reliable selection tool; high methodological robustness compared with other MCDM approaches.	High computational complexity; dependence on accurate and comprehensive data; results tailored to specific configurations, limiting replicability.
ID_18	Enhanced decision-making quality; flexibility for adaptation to other contexts; ability to identify balanced and effective solutions by considering multiple criteria.	Increased methodological complexity; requirement for high-quality data; need for specialized knowledge to apply the techniques.

Source: Authors (2026). * Identifying the items within the portfolio.

**Table 5:** Sectors represented in the refined articles of the analysis portfolio.

ID *	Sector
ID_1	Business and Management
ID_2 and ID_9	Health
ID_4	Education
ID_13	Alternative Energy
ID_18	Civil Engineering

Source: Authors (2026). * Identifying the items within the portfolio.

**Table 6:** Authors’ suggestions for future research directions.

ID *	Authors’ Suggestions
ID_1	Conduct sensitivity analyses to evaluate how changes in weight values affect the final ranking of alternatives; identify the most critical criteria; evaluate the robustness of the classification results.
ID_2	Develop faster, more streamlined versions of the tools for initial prioritization; expand the pathogen scope using international databases; consider separate assessments for probability of emergence and impact when suitable; apply methods to quantify expert uncertainty (e.g., Cooke’s method, Bayesian networks); explore ways to translate scores into practical recommendations; integrate climate variables into the modeling framework.
ID_4	Apply different multicriteria methods in parallel to increase the reliability of results; develop automated decision-support tools; extend the model to other application domains; investigate the effectiveness of the decisions made when the model is implemented in real-world practice.
ID_13	Extend the method to other renewable energy systems; explore combinations with emerging and improved technologies; validate the approach through practical tests and real-world implementations.
ID_18	Apply the method in other contexts; develop automated tools to integrate multicriteria methods; validate the proposed approach through detailed case studies.

Source: Authors (2026). * Identifying the items within the portfolio.

## Data Availability

No new data were created or analyzed in this study. Data sharing is not applicable to this article.
